# Freezing of Gait in Parkinson’s Disease: A Scoping Review on the Path Towards Real-Time Therapies

**DOI:** 10.3390/s26072042

**Published:** 2026-03-25

**Authors:** Meenakshi Singhal, Christina Grannie, Margaret Burnette, Manuel E. Hernandez, Samar A. Hegazy

**Affiliations:** 1 Carle Illinois College of Medicine, University of Illinois at Urbana-Champaign, 506 South Mathews Avenue, Urbana, IL 61801, USA; ms189@illinois.edu (M.S.); grannie2@illinois.edu (C.G.); phburn@illinois.edu (M.B.); 2 Department of Bioengineering, University of Illinois at Urbana-Champaign, 506 South Mathews Avenue, Urbana, IL 61801, USA; 3 Beckman Institute for Science and Technology, University of Illinois at Urbana-Champaign, 506 South Mathews Avenue, Urbana, IL 61801, USA

**Keywords:** closed loop, machine learning, Parkinson’s disease, freezing of gait

## Abstract

Background: Freezing of gait (FoG) is a common symptom of Parkinson’s disease, especially in its later stages of progression. Characterized by involuntary stopping during normal gait patterns, FoG greatly increases fall risk, reducing quality of life. Given the complex presentation and etiology of FoG, current treatments have proven ineffective in managing episodes. In recent years, machine learning algorithms have been leveraged to derive actionable clinical insights from biomedical datasets. As a manifestation of neuromechanical dysfunction, impending FoG episodes may be characterized through data collected by wearable devices and sensors. Objective: This scoping review evaluates the current landscape of machine and deep learning-derived biomarkers to enhance the personalized management of FoG. Methods: This scoping review was conducted using established methodological frameworks for scoping reviews and is reported in accordance using the PRISMA-ScR checklist. Three databases were queried, with screening yielding 60 studies. Results: Thirty-nine papers reported on deep learning techniques, with the most common architectures being convolutional neural networks and long short-term memory models. Conclusions: Inertial measurement units, which can be worn on various locations, may be a promising modality for practical implementation. To generate closed-loop FoG therapies, algorithms can be integrated into real-time systems like robotic exoskeletons or adaptive deep brain stimulation. Future work in generating datasets from ambulatory devices, as well as distributed computing strategies, may lead to real-time FoG management.

## 1. Introduction

Parkinson’s disease (PD) is a neurodegenerative movement disorder affecting over 1 million people in the U.S. and 10 million worldwide [1]. Clinical features include both motor and non-motor symptoms, such as sleep disturbances, fatigue, depression, anxiety, and apathy. Motor symptoms include tremors, shuffling, bradykinesia, postural instability, and rigidity, which can all drastically reduce quality of life when inadequately managed. In particular, freezing of gait (FoG) is a phenomenon where patients temporarily lose the ability to move their legs forward while walking. Although FoG is most associated with advanced disease progression, it has been reported in approximately 40% of the overall Parkinson’s disease population and in more than 70% of patients with a disease duration of 10 years or longer [2]. Often triggered by sudden movement changes like turning, FoG episodes significantly increase the risk of falls, making them a critical area within PD. A major clinical challenge in FoG management is its variable presentation across patients. While some may experience gait freezing when they first initiate movement from rest, others may find they are more susceptible to episodes while dual-tasking, such as both speaking and walking. Moreover, differences in the nature of festination, i.e., foot shuffling, that patients express prior to freezing create another parameter of consideration [3]. Standard therapies, including levodopa regimens [3] and continuous deep brain stimulation (DBS) [4], have demonstrated limited efficacy in mitigating symptoms. Certain patients may be more susceptible to FoG during medication ON state, OFF state, or transition phases between medication states; meanwhile, there may be a trade-off between gait-optimized DBS frequency and appendicular side effects [5]. Common triggers include turning, doorway/narrow-space negotiation, and dual-tasking, contributing to between-patient variability in freezing phenotypes.

While the underpinnings of FoG remain an area of active study, the neuromechanical nature of its presentation lends itself to a variety of analytical techniques. The rise in wearable sensor technologies within healthcare has facilitated a revolution of continuous monitoring, rehabilitation, and other applications that leverage data-driven approaches. In particular, the development of digital biomarkers derived from inertial measurement units (IMUs) has enabled improved management of neuromuscular conditions, including stroke [6,7] and muscular dystrophy assessment [8].

**Figure 1:**
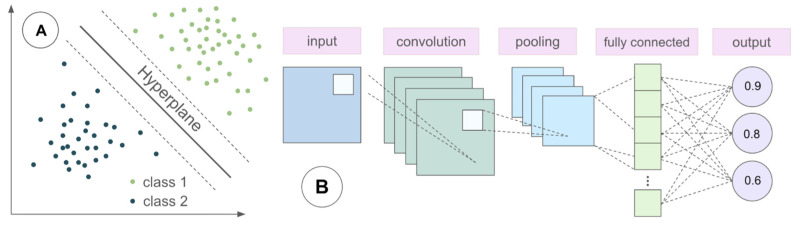
Representation of (**A**) support vector machine, which optimizes the position of a decision boundary called the hyperplane to perform classification and regression tasks; (**B**) CNN, whose parameters (e.g., layer number and type, filtering, activation functions) can be highly customized.

Machine learning (ML) is a subset within the larger field of artificial intelligence (AI), with algorithms allowing for prediction and decision-making by learning trends from given data in a supervised manner. Traditional ML algorithms include linear regression, support vector machines (SVMs, Figure 1A), k-nearest neighbors (kNN), tree-based methods, and the naive Bayes classifier. While each model processes information in a different manner, they all train by making decisions on pre-labeled data. Deep learning (DL) is a more specialized subset of ML in which neural network models can infer underlying trends from unlabeled data, optimizing their own actions through an unsupervised learning process. While ML algorithm performance may plateau, DL performance tends to increase alongside data input. By combining multiple sets of hidden layers and weight functions, convolutional neural networks (CNNs, Figure 1B) and other DL architectures offer extensive flexibility for model optimization. Although DL algorithms are defined by their ability to provide pattern recognition insights in large-scale, complex datasets, there are tradeoffs in terms of training time and feature interpretability.

By training on transcriptomic, imaging, and other data sources, ML/DL has powered the discovery of biomarkers in cancer [9], neurological conditions [10], and diabetes [11]. At the time of writing, upwards of 80% of AI algorithms currently approved by the U.S. Food and Drug Administration are within radiology [12], highlighting the ease of data acquisition in the field. For freezing of gait, recent data science contests suggest a community-wide push towards establishing clinically relevant AI models [13]. Identifying objective markers for FoG is the first step towards developing closed-loop, real-time therapies. To this end, we conducted a scoping review to map the current progress and challenges in applying ML techniques to datasets, towards enabling data-driven methods to improve the management of FoG in patients with Parkinson’s disease.

## 2. Materials and Methods

This scoping review was conducted using established methodological frameworks for scoping reviews and is reported in accordance with the PRISMA-ScR guidelines (Supplementary Materials) [14]. The PubMed, Web of Science, and SCOPUS databases were queried on 4 February 2025, with a curated search strategy, with the PubMed version provided:

((artificial intelligence[majr:noexp] OR machine learning[majr:noexp] OR deep learning[majr:noexp] OR neural networks, computer[majr:noexp] OR (artificial intelligence[tiab] OR machine learning[tiab] OR deep learning[tiab] OR neural networks[tiab])) AND (Parkinson disease[MeSH] OR “Parkinson’s disease”[tiab] OR “Parkinson disease”[tiab])) AND ((“freezing of gait”[tiab]))

**Figure 2:**
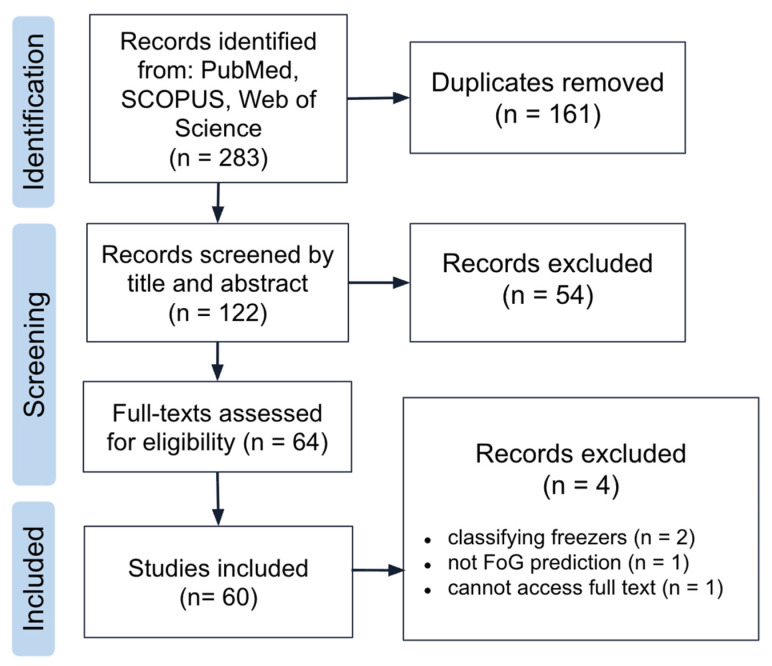
PRISMA diagram depicting study selection process.

This search strategy yielded 122 unique results (Figure 2). Peer-reviewed papers in the English language were included if they: (1) were published between 2020 and 2025; (2) address patients diagnosed with PD; (3) focus on predicting, detecting, or treating FoG episodes; (4) involve the application of machine learning algorithms to process data; and (5) include analyses of datasets based on electrophysiological and/or biomechanical data. Given the rapid advancements in machine learning within healthcare and wearable devices, only papers from the given time range were included. While literature may have explored FoG within a broader analysis of mobility and Parkinson’s disease, those papers may not have been represented within the results, as the focus of the search query was on FoG. Exclusion criteria included editorials, review papers, book chapters, animal model studies, data science competitions or other formats not exploring the work generated by a single team in depth, or works not focused on the management of FoG. The full dataset independently underwent title and abstract screening by two reviewers (M.S. and C.G.), and discrepancies were resolved through discussion. Full-text review on the resulting 64 studies was split between both reviewers, who held joint discussion on decisions for all papers. Following full-text screening, 60 papers underwent data extraction.

## 3. Results

In general, FoG management was explored as either episode prediction, detection, or therapeutic intervention within the 60 included studies (Table 1). In addition to performance measures like sensitivity, specificity, and AUC, domain-specific metrics such as latency time and computational complexity were frequently assessed to evaluate the translational potential of a given algorithm.

### 3.1. Data Modalities, Features, and Model Performances

**Figure 3:**
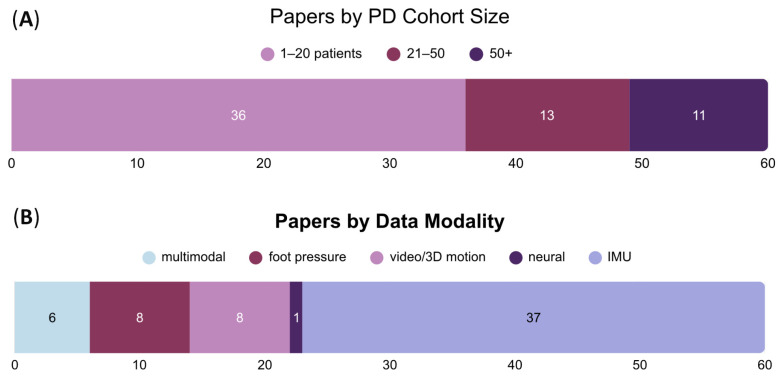
Papers stratified by: (**A**) number of PD patients included in dataset; (**B**) type of training data, with multimodal including combinations of several forms.

The models in the included studies were trained on several key categories of data from patients with Parkinson’s disease. Thirty-seven papers utilized inertial measurement unit (IMU) recordings containing accelerometry (and occasionally gyroscopic and/or magnetometry) data taken from various locations on the body (Figure 3). Table 2 further explores the variability and trends in IMU positioning. Anywhere between one and seven sensors were placed on participating patients, with the optimal combination and number differing across studies. Several papers have demonstrated that sensor count does not correlate with higher model performance for FoG or other gait-related conditions. Rather, sensor positioning plays a larger role in effective mobility assessment and long-term monitoring [39,75,76]. From a translational perspective, the authors note that certain locations, such as the ankle, may be more feasible for incorporation within a closed-loop wearable for FoG reduction. Key considerations include noise, patient preference, and anatomical variability. Overall, the most common IMU placement was at the ankle, followed by the hips and knees.

Some groups found that incorporating wavelet-based features was particularly beneficial for model training time and performance. With analytical methods such as the Fast Fourier transform, time-domain data can be translated into the frequency domain, revealing new temporal information. Noting the heterogeneity in FoG presentation across patients, Shi et al. [41] aimed to unite the two domains by generating time-frequency wavelet representations. The researchers converted raw time-series IMU recordings from 67 PD patients into Morlet continuous wavelet transforms, achieving an F-1 score of 91.5% using an 8-layer CNN architecture. Meanwhile, eight papers involved plantar pressure data collected from wearable insole sensors or force plates. Taking advantage of the cyclical nature of overground walking, researchers harnessed features like ground reaction forces. In general, models trained on video or 3D motion capture data (*n* = 8) had lower performance across both ML and DL architectures, with accuracies in the 70–80% range.

While kinematic features provide insight on the biomechanical component of FoG, its onset as dysregulation within basal ganglia circuitry allows neurophysiological information to lend another perspective. One study incorporated solely neural data in the form of electrocorticography (ECoG) recordings taken from patients with DBS implants. Liu et al. [22] found that beta (13–30 Hz) power in the subthalamic nucleus (STN) was significantly higher during FoG episodes than in normal walking, with minimal variability throughout standard walking periods. The researchers allude to modifying DBS algorithms to target FoG based on such gait-specific patterns, a concept further explored in the Discussion. Six papers employed a combination of multiple data streams, such as electroencephalography (EEG) in tandem with electromyography (EMG). Li et al. [68] proposed a form of multimodal fusion with adaptive weighting, integrating IMU and insole sensor data. The group noted that their “feature-fusion-weighted” model outperformed models trained on a single data modality.

### 3.2. Dataset Diversity and Augmentation

To address concerns related to dataset quality, papers incorporated various data augmentation strategies. Thirty-seven of the sixty studies trained on datasets from cohorts of twenty or fewer PD patients (Figure 3); 14 of these utilized the publicly available Daphnet Freezing of Gait dataset. Daphnet, curated in 2013, contains 237 FoG events recorded from ten patients diagnosed with Parkinson’s disease, with an average age of 66.5 years. A total of 8 h and 20 min of recordings are included from three triaxial accelerometry sensors (ankle, hip, and knee) worn during both assigned walking tasks and activities of daily living (ADLs) [77]. Hinting at the need for more diverse and thorough datasets in this space, one study focused exclusively on drawing from generative artificial intelligence (genAI) techniques to create a novel dataset. By training a generative adversarial network on Daphnet, Peppes et al. [66] created a synthetic FoG dataset that, when validated with a deep neural network, achieved 92.09% accuracy in FoG episode classification.

Given the challenges of collecting ambulatory data, patients were generally recorded within a standardized clinic setting as opposed to an at-home environment. Thus, for the majority of studies, FoG episodes were “induced” by various exercises like the Timed Up and Go (TUG) mobility assessment or dual-tasking. In line with this trend, a considerable number of datasets were highly imbalanced, with a relative scarcity of FoG episodes relative to the total recording time. Al-Nefaie et al. [18] and Mostafa et al. [44] address this discrepancy through the “synthetic minority oversampling strategy” (SMOTE), a widely used resampling method. SMOTE functions by sampling along temporary delineations between data points in the minority class (e.g., FoG episodes) that are close in proximity within the feature space [78]. While the three teams built models with notably high performance on FoG prediction tasks (Table 1), oversampling and genAI methods raise broader questions about the translation of algorithms trained on data patterns not present in the real world.

### 3.3. Algorithmic Design and Parameters

**Figure 4:**
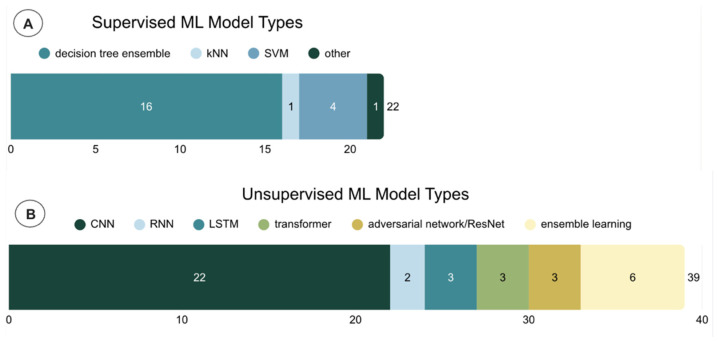
Papers stratified by: (**A**) supervised ML algorithm; (**B**) unsupervised ML algorithm. Ensemble learning models involved CNNs combined with another type.

**Figure 5:**
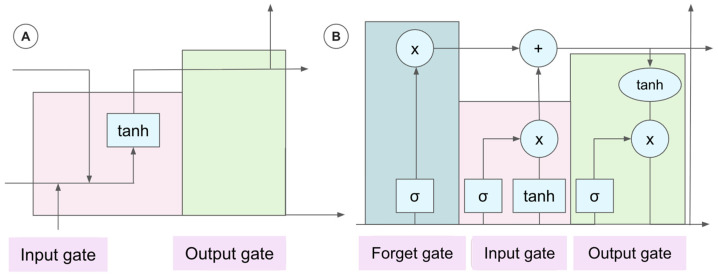
Representation of (**A**) recurrent neural network (RNN); (**B**) long short-term memory model (LSTM). LSTMs build upon the standard RNN by incorporating an additional “forget gate”. This enables selective information retention, allowing the model to more efficiently learn long-term dependencies.

For the 22 papers based on conventional ML algorithms, the SVM most frequently had the highest performance, followed by kNN. Though decision tree ensembles like random forest, XGBoost, and AdaBoost were the most common supervised model category, they typically presented with lower metric scores. For these supervised ML papers, ground truth was usually established by clinician-labeled FoG episodes. Thirty-nine of sixty papers applied deep learning models (Figure 4) to FoG datasets; of these studies, three found long short-term memory (LSTM) models to be the most effective, and the rest reported on another form of recurrent neural network (RNN), transformer, adversarial neural network, or CNN. In general, RNNs (Figure 5) function to process sequential data with built-in feedback loops, making them well-suited for time-series analysis. LSTMs build upon this framework through an added memory gating system (Figure 5).

### 3.4. Strategies for Improved Performance

Studies utilizing DL algorithms applied several strategies to improve model performance. Six papers employed ensemble learning, which combines two or more architectures to leverage their individual strengths. Each ensemble contained a CNN in conjunction with either an LSTM or RNN, allowing for multifunctional benefits. While CNNs are generally suited for spatial data, LSTMs can provide insights into time-dependent trends. Indeed, the performance of all ensemble classifiers was in the 95–99+% range, except for one that trained on video recordings.

**Figure 6:**
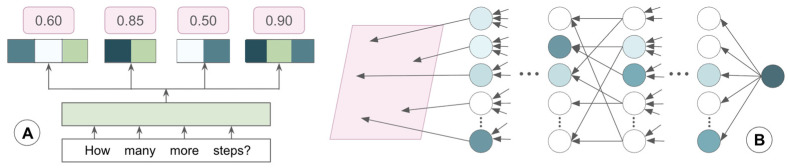
Representation of (**A**) attention mechanism, utilized to help direct DL models towards the most relevant information; (**B**) layer-wise relevance propagation, which involves decomposing model outputs to derive the most meaningful input features.

Meanwhile, teams focusing on LSTMs found that adding an attention mechanism (Figure 6) into the architecture enhanced FoG episode detection. By assigning greater weights to certain features, an attention mechanism boosts a model’s ability to capture large-scale relationships and process complex time series data, as in the case of sequential recordings containing FoG episodes. The squeeze-and-excite block, one form of attention mechanism, offers versatile integration into DL architectures with minimal added computational cost. In the 60 studies, it was incorporated within both LSTMs and CNNs and could be separated into three components: (1) squeeze; (2) excite; (3) scale. During the first phase, global average pooling condenses the fully connected layer into a single embedding, improving generalizability on new data. In the latter phases, the channels are recalibrated based on the simplified embeddings [79]. CNNs were also augmented to address interpretability: Filtjens et al. [40] utilized layer-wise relevance propagation (Figure 6), an Explainable Artificial Intelligence method, to explore the most salient joint trajectory features taken from 3D motion capture software.

### 3.5. Closed-Loop Systems

Ultimately, the clinical management of FoG depends on therapeutic intervention in addition to episode detection. Six papers demonstrate “closed-loop” systems that aim to do so. In 2023, Koltermann et al. [70] published FoG-Finder, which functions with a 615 ms latency period between FoG episode prediction and cueing. FoG-Finder implements a CNN architecture trained on ankle accelerometry data to time the delivery of vibrotactile stimulation based on detected FoG episodes. Koltermann et al. [16] went on to develop Gait-Guard, which once more provides vibrotactile cueing based on ankle IMU recordings, now processed by a transformer DL model. The authors note that Gait-Guard had an improved latency period of 378.5 ms, suggesting real-world benefit to affected patients and the potential for architecture optimization to improve performance. Dvorani et al. [19] emphasize their advances in edge computing, a distributed model where data processing occurs along multiple nodes (e.g., sensors) that are closer to the user, rather than in a centralized location. This methodology enabled researchers to obtain a latency of 7.5 ms between sensor recording and electrical stimulation. Hou et al. [35] implemented multimodal prediction based on signals from a custom around-the-ear wearable EEG and flexible sensors capable of recording EMG and accelerometry signals from the calves and shins. When an impending FoG episode is detected by a pretrained CNN classifier powered by an integrated circuit, an auditory cue is delivered to the patient via a speaker. Although latency values are not reported, the researchers highlight the low power consumption of the hardware, as well as the model’s high AUC (0.85).

## 4. Discussion

**Figure 7:**
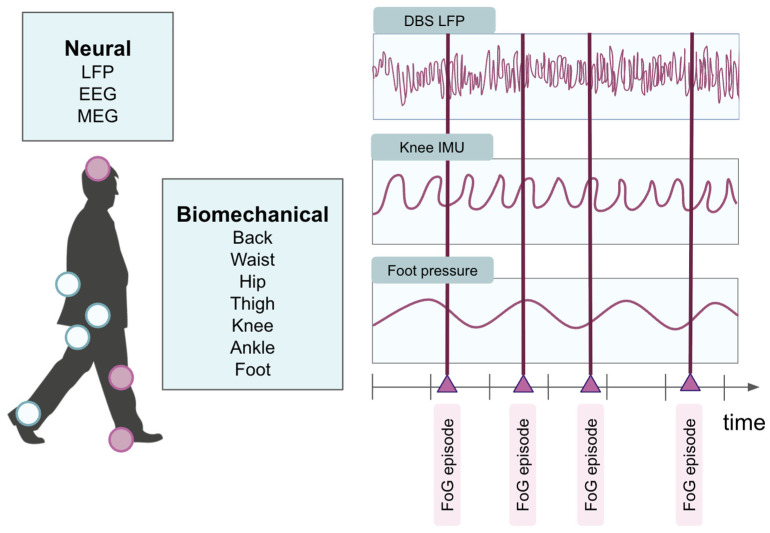
Example sensor locations on the body for ambulatory data collection during walking. White circles indicate representative anatomical sites where biomechanical and neural signals may be recorded, including the back, waist, thigh, and ankle. Pink highlighted circles represent intracranial DBS electrodes (local field potentials), a knee-mounted inertial measurement unit (IMU), and insole pressure sensors at the feet. Time-synchronized neural and biomechanical signals during walking and freezing-of-gait (FoG) episodes can be used to derive biomarkers that inform personalized, closed-loop therapeutic algorithms.

The variety of AI models that researchers tested emphasizes the clinical challenge presented by FoG. Across the included studies, DL methods demonstrated improved performance compared to traditional ML models, which may be in part due to the complex nature of overground walking. While FoG can be modeled as a binary classification problem, the episode is preceded by a series of gait events, like shuffling or trembling, that contain information about underlying disease progression, pathophysiology, and other nonlinear features that DL approaches may be more amenable towards detecting. In particular, LSTMs, which can capture trends in sequential data, could be an ideal baseline architecture to explore and augment for FoG management. The results of this review paper also suggest that IMUs are a promising modality for detecting FoG episodes, as both ML and DL models built upon accelerometry data consistently performed well (Table 1). Of note, the straightforward nature of sensor placement and removal would facilitate data collection and quality, allowing for greater accessibility across the PD patient population. While the vast majority of included studies report exclusively on predictive algorithms, detection is only the first step of FoG episode management. To effectively “close the loop” and improve patients’ quality of life, a therapeutic aspect (Figure 7) must follow.

### 4.1. Closed-Loop Neuromodulation

The closed-loop systems explored in the Results all provide intervention in the form of tactile or auditory stimulation to the user. As FoG is inherently a manifestation of a neuromechanical disorder, exploring this realm may confer more physiologically relevant therapy. In February 2025, Medtronic (Minneapolis, MN) received approval from the U.S. Food and Drug Administration to implement adaptive deep brain stimulation (aDBS) algorithms on the Percept™ PC device to treat motor symptoms in patients with Parkinson’s disease. The advent of sensing-and-stimulating, implantable hardware like Percept™ PC enables at-home electrophysiological data to be streamed and analyzed for biomarkers of pathological state, which can then be programmed back within the device. Unlike continuous DBS, which can exacerbate FoG by delivering constant stimulation irrespective of neural state, aDBS would dynamically adjust stimulation in real time based on specific neural signatures. In a landmark study, Oehrn et al. [80] demonstrate the long-term feasibility of aDBS in four patients by linking stimulation to local field potential (LFP)-derived control signals. To generate the biomarkers, the team applied neural recordings into a linear discriminant analysis classifier, a form of supervised learning. While FoG episodes were not specifically addressed by the study, the researchers tailored each patient’s neural biomarker to their individual motor symptom profile, such as bradykinesia or dyskinesia.

Based on this framework, the unique neural signatures expressed during movement suggest considerable potential to personalize the algorithms used in aDBS to treat FoG. By synchronizing kinematic and LFP recordings from the Medtronic Summit RC+S device, one group identified pathological beta frequency (13–30 Hz) bursts in the STN as a candidate control signal for mitigating FoG in seven patients [81]. In addition to reducing FoG episode duration and frequency, the aDBS paradigm was shown to improve bradykinesia, walking tasks, and Unified Parkinson’s Disease Rating Scale (MDS-UPDRS III) scores. However, when the researchers compared outcomes with adaptive and traditional DBS, they found minimal differences, which they posit may be due to the need for a longer acclimation period for the newer paradigm.

The extent to which a singular neural signature (e.g., beta bursts) can encapsulate underlying FoG pathophysiology is likely to fluctuate based on environmental factors [82]. Looking ahead, the discrepancies in clinical presentation (freezing while turning, start hesitation, etc.), the role of emotional processing, and the breadth of algorithmic control policies each warrant resolution when developing aDBS strategies for FoG [83,84]. Although all aDBS studies reported in this paper harnessed supervised learning techniques for biomarker discovery, future systems may incorporate a DL approach that automatically adjusts the neural control signal over time to account for temporal changes. However, adjusting stimulation settings with individual gait cycles means adapting at relatively short-term durations (i.e., milliseconds), when events occurring on longer timescales, like cognitive cues, may also confer an effect. Advances in reinforcement learning and brain state decoding could pave the way for algorithms that adapt across multiple timescales, from the FoG episode itself to more longitudinal measures like disease progression. For example, Horn et al. [85] propose the potential for aDBS algorithms to automatically switch between treating different PD symptom states, like tremor and rigidity, by decoding at the brain circuitry level. Termed the “adaptive circuit targeting” DBS framework, their system would incorporate connectome information to attain a more naturalistic form of therapy. Ultimately, a stronger mechanistic understanding of FoG will better inform how neuromodulation can be harnessed to manage or even prevent episodes.

### 4.2. Closed-Loop Biomechanics

Despite algorithmic advances being important for accurate FoG prediction, the translation of these models into the real world as therapeutic tools depends on their biomechanical implementation. External devices such as robotic exoskeletons, soft robotic apparel, and insole-based systems have been explored as potential additions to ML-driven FoG detection [86,87]. These devices aim to provide mechanical cues or gait compensation during FoG episodes, thereby reducing fall risk. However, their effectiveness is limited by various biomechanical considerations.

Any wearable device must maintain the body’s natural gait mechanics. Exoskeletons, for example, may stabilize joint motion but can also inadvertently alter arm swing, stride length, or add in compensatory movements, potentially creating maladaptive gait patterns over time [88]. Similarly, insole-based sensors show promise as a minimally invasive option, but prolonged use could affect comfort, weight distribution, and plantar pressure, thereby affecting gait [89]. In comparison, soft robotic garments offer improved flexibility and adaptability compared to rigid exoskeletons, but they still have to deliver precise torques to specific joints without interfering with natural movements, often incorporating cable actuators to augment hip flexion to combat FoG episodes [87].

Furthermore, patients may consciously alter the way they walk because they are aware of the device, or unconsciously compensate for added bulk, weight, or poorly positioned sensors [88]. These compensations could shift loading patterns across joints, muscles, and bones in ways that, over time, could lead to secondary musculoskeletal issues. For example, an insole that relieves FoG in the short term might subtly alter knee or hip biomechanics, increasing strain on proximal joints [90]. Likewise, heavier or inflexible devices could affect balance, posture, or arm swing [88]. These risks underscore the need for ergonomic, lightweight, and unobtrusive device designs, as well as longitudinal studies that evaluate both FoG reduction and longitudinal consequences on joint health and overall musculoskeletal function.

Another challenge is in patient-specific biomechanics. Anatomical differences, disease states, and comorbidities influence how wearables interact with patients. For example, stride variability, muscle strength, and foot anatomy can alter the effectiveness of interventions [91]. Adaptive algorithms tailored to each patient’s biomechanics could help personalize therapy while minimizing unintended gait disruptions. This emphasizes the need for future directions in which wearable devices serve as both a diagnostic tool and as a device for individualized therapy and treatment that is based on the anatomy of each patient.

Lastly, practical factors such as device comfort and integration into daily life are critical for implementing wearables in the real world. Real-world feasibility requires patients to tolerate these medical devices for long durations without interfering with ADLs. Translating prediction into effective closed-loop interventions will therefore require careful design of wearable hardware to ensure that biomechanical considerations do not undermine therapeutic benefit, and vice versa.

### 4.3. Translational Considerations Towards Real-Time Therapies

Translating predictive architectures into meaningful therapies will require resolving several data-related challenges. While sophisticated algorithms may offer a more accurate prediction of upcoming FoG events, this trend creates a notable tradeoff between model complexity and robustness. An improvement in traditional metrics may not directly correlate with real-world application due to the technological constraints of wearable and implantable devices. Complex architectures incorporating elements like ensemble learning may require greater power consumption, making battery life a potential concern. In addition, the latency period between FoG detection and intervention should be within a certain range; otherwise, therapy (i.e., neurostimulation, torque forces, cueing) will not be delivered at clinically relevant timescales. For example, if aDBS algorithms modulate stimulation parameters at the gait-cycle level, the adjustment period of amplitude ramping may not align with the FoG episode, reducing efficacy.

A limitation that spread across the included studies was relatively small participant cohorts. It is imperative that this limitation be taken into account when interpreting deep learning results. These models tend to perform better when they are trained on large datasets. Therefore, smaller sample sizes may increase the risk that performance reflects overfitting rather than true FoG features. Furthermore, the persistent reuse of the same public datasets, along with not testing enough on independent data, can inflate the reported accuracy. This diminishes confidence in how well these approaches may actually translate to real-world settings [92].

To better separate true FoG features from differences between patient groups, future studies should analyze results by medication status and task conditions (e.g., turning or dual-task walking), clearly report disease severity and duration, and use study designs or analysis approaches that account for differences between individuals.

Because most of the studies used cohorts with 20 or fewer participants and because many papers utilized the same or similar datasets, e.g., Daphnet, the performance improvements reported may capture dataset-specific patterns rather than true freezing-of-gait physiology; this is especially the case with complex DL models. At a minimum, we suggest that future studies test the models on new patients, try to use independent datasets, and lastly, report practical performance details (the number of detected episodes, response timing, and energy use, for example) in conjunction with the standard accuracy scores to avoid reporting potentially overly optimistic conclusions.

Towards addressing these concerns, the rise in the “Internet of Things” across healthcare and various industries has ushered in a new wave of improvements in data processing efficiency. As explored in Section 3.5, intentional algorithmic design choices can greatly enhance the translational potential of models. Dvorani et al. [19] demonstrate that edge computing can reduce latency and maximize computational resources; delegating calculations to a distributed network of sensors may thus be a valuable strategy for deploying closed-loop systems, especially in biomechanical solutions such as exoskeletons. Moreover, while the DL models in this study tended to provide greater performance than their traditional ML counterparts, they also require considerably more data for training. Ongoing advances in personalized algorithm design can help optimize real-world scalability and also address the patient-specific variability of FoG. Transfer learning is one computational approach that could balance the need for individualized models with the aforementioned constraints. The method involves first training a foundational AI model to solve a given task (e.g., FoG management in Patient #1), which can later be reapplied to solve related tasks (e.g., Patient #2). Transfer learning operates on the principle that there is a generalizable basis across tasks or patients, such that initially learned features are applicable to novel scenarios. Rather than expend extensive resources to collect FoG episode data and build a model from scratch for each patient, pre-trained transformer models can be finetuned with smaller datasets [93,94], reducing computational, patient, and healthcare provider burden.

Finally, taking into consideration prior insights on IMUs (Table 2), optimizing the location of fewer total sensors can reduce model complexity, while still retaining high performance. In particular, O’Day et al. found that on a FoG prediction task, a single ankle IMU had similar performance (AUC = 0.80) to the best combination of IMUs in the study, which was the lower back and both ankles (AUC = 0.83) [39]. We see similar examples of this trend in the included studies: Chan et al. found that a CNN trained on a single IMU placed at the waist had 83% accuracy [17]. Meanwhile, Yang et al. trained a temporal CNN on data from five IMUs on the pelvis and lower body, which conferred an F1-score of 0.63 [30]. However, direct comparison across studies remains challenging due to differences in patient populations, model architectures, and the nature of reported metrics. Future research studies focused on IMU optimization and patient preferences can improve the real-world translation of data-driven insights.

### 4.4. Limitations

This review highlights several limitations in the current landscape of machine learning applications for FoG management. First, there was substantial heterogeneity in reported performance metrics. Whereas some studies emphasized only the accuracy or F1-score, others focused on sensitivity, specificity, or AUC, making direct comparisons across models challenging. Moreover, due to the varying influence of class imbalance on each metric, the one that a given paper may have optimized its model on could directly affect its translational potential. With this in mind, standardized reporting frameworks could better facilitate proper benchmarking and reproducibility. Of note, the closed-loop papers discussed in Section 3.5 did not utilize the number of falls as an outcome measure, despite its notable relevance to the goals of FoG reduction. Implementing more clinically meaningful metrics like fall reduction, real-time power consumption, and patient comfort will be important considerations for validating future closed-loop devices.

Second, the size and diversity of study cohorts were limited. A majority of the studies had fewer than 20 patients, and many drew from the same publicly available datasets, such as the Daphnet Freezing of Gait dataset. Small cohort sizes were consistently a limitation across the studies included in this review. This is particularly problematic with the use of deep learning. Deep learning models often require large and diverse datasets to establish stable and generalizable representations. Thus, training on limited samples, repeated use of publicly available datasets, and limited testing on independent external datasets may contribute to high performance estimates and diminished generalizability to the real world [92]. These constraints are further influenced by clinical heterogeneity both within and across study populations. Variabilities in age, disease duration and severity, and medication status are known to influence the occurrence and presentation of FoG, yet these factors are inconsistently accounted for, which limits the model interpretability and translational relevance. Having small and homogeneous samples limits the generalizability of findings, specifically when one considers the variability in FoG presentation across disease states, demographics, and comorbidities. While each included paper reported on the patient cohort size, the vast majority did not provide statistics regarding the total count of individual labeled FoG episodes, which would provide more granular information on dataset quality. In addition, the motor impairment severity of patients was rarely reported, and usually not in a standardized manner, i.e., MDS-UPDRS III scores. Improving data collection to include larger and more diverse populations could help ensure that algorithms maximize external validity to real-world settings. When the number was reported, FoG episodes were often relatively scarce compared to the total duration of gait recordings, leading to overrepresentation of normal walking patterns. The usage of data augmentation strategies like synthetic oversampling and generative AI in the resulting studies warrants further evaluation, stressing the need for more nuanced metrics to evaluate model performance. Models built with these features may present with high accuracy in a computational setting, but may not take into account contextual and behavioral factors associated with mobility. While synthetic oversampling methods like SMOTE were applied in some cases, these approaches cannot entirely substitute for high-quality, naturally balanced data. Without substantive external validation, it remains uncertain whether these approaches to handling imbalanced biomedical data truly augment performance. More extensive long-term monitoring could help capture a better distribution of FoG events.

Additionally, the data collection environment was underreported and varied considerably across studies, with most recordings conducted in controlled clinical settings where FoG was induced by standardized tasks (e.g., TUG). While these environments ensure consistent labeling, they may not reflect the complexity of FoG in day-to-day life. The reliance on induced FoG using standardized clinical tests, such as the Timed Up and Go and dual task test, raises important questions about the ecological validity of the reported findings and generalization to at-home settings or nursing homes [95,96]. Ambulatory, at-home monitoring is needed to capture more naturalistic episodes and environments, which will be critical for building algorithms that are adaptable to real-life scenarios and true to each patient’s needs and space. These limitations highlight the necessity for more comprehensive, real-world datasets and standardized evaluation practices. Addressing these gaps will be essential for advancing ML- and DL-based FoG detection into reliable, clinically useful tools.

Finally, although this review emphasized real-time detection and closed-loop therapeutic applications, only a small minority of the included studies implemented a true “closed-loop” structure. Key practical considerations for real-world employment (detection latency, computational burden, clinically meaningful outcomes, and fall reduction) were inconsistently reported. This observation showcases a gap between the current translational goals in a clinical setting and the available evidence in the literature. As such, our statements regarding real-time and closed-loop feasibility should be interpreted as hypothesis-generating rather than confirmatory.

In addition to the limitations within the reviewed literature, this review itself also comes with its own limitations. Firstly, the search strategy employed in this review focused on studies that referenced “freezing of gait,” specifically. This confined search strategy may have led to the exclusion of relevant works that included FoG characteristics without explicitly using that terminology. This restriction may have influenced the scope and representativeness of the included literature and should be considered when interpreting the findings of this review.

## 5. Conclusions

As FoG is a major contributor to the number of falls that a patient with PD may experience—and falls are closely tied to increased risk of injury and hospitalization—it is critical to establish accurate detection and prediction systems that improve patient safety. This review highlights both the progress and the challenges that ML and DL methods provide within sensor-based FoG management. These high-throughput approaches can be applied to identify FoG episode biomarkers and generate therapies in a personalized manner. In particular, IMUs and algorithms such as CNNs and LSTMs consistently demonstrated strong performance, emphasizing the potential for an objective gait monitoring framework based on these combined techniques.

However, this review also shows that translation into real-world practice and the use of closed-loop therapies remain limited. As only a small fraction of the included studies utilize a closed-loop framework, practical challenges to implementation may not yet have been uncovered or resolved. Obstacles in contemporary research include the use of small, homogeneous datasets, inconsistent reporting standards, and the need to integrate biomechanical and neurophysiological aspects. Future work should prioritize the adoption of more diverse, naturalistic datasets and distributed computing strategies that optimize therapeutic parameters in real time, as well as clinically relevant measures like fall reduction and latency. Looking forward, physiology-driven devices that leverage ML could offer valuable benefits for patients with PD, including reduced FoG, reduced fall risk, and improved mobility and quality of life.

## Figures and Tables

**Table 1:** Characterization of *n* = 60 included studies; AUC = area under the curve, ECoG = electrocorticography, CNN = convolutional neural network, GBM = gradient boosting machine, IMU = inertial measurement unit, kNN = k nearest neighbor, PD = Parkinson’s disease, RNN = recurrent neural network, TCN = temporal convolutional network.

Author	Patient Population	Machine Learning	Data Modalities	Model Type and Features	Performance Metrics
Slemenšek et al. [15]	6 PD patients	deep learning	accelerometer, gyroscope, and muscle activity sensors	CNN + RNN	95.1% accuracy, 98.8% specificity, 2.3% precision, 2.4% sensitivity
Koltermann et al. [16]	26 PD patients	deep learning	2 IMUs (L/R ankles)	multivariate time-series transformer encoder	96.5% accuracy
Chan et al. [17]	107 PD patients	deep learning	1 IMU (waist)	CNN	83% accuracy, 89% sensitivity, 81% specificity
Al-Nefaie et al. [18]	65 PD patients	machine learning	1 IMU (lower back)	decision tree	91% accuracy, 97% precision, 99% sensitivity, 0.94 F1-score
Dvorani et al. [19]	16 PD patients	machine learning	1 IMU (foot)	AdaBoost	0.875 ± 0.082 AUC, 84.0% ± 7.0% accuracy, 85.5% ± 8.6% sensitivity, 78.8% ± 11.9% specificity
Li et al. [20]	50 PD patients	machine learning	Mobile phone video recordings (OpenPose annotations)	XGBoost	81.56% accuracy, 87.5% sensitivity, 79.82% specificity
Mesin et al. [21]	12 PD patients	machine learning	2 IMUs (L tibia and wrist), and EEG	Support vector machine	88% accuracy, 85.14% sensitivity, 88.38% specificity, 87.71% precision, 0.8673 F1-score
Liu et al. [22]	8 PD patients	machine learning	ECoG and local field potentials	mixed-effects random forest	77% accuracy, AUC: 0.75
Borzi et al. [23]	11 PD patients	machine learning	2 IMUs (L/R shins)	Support vector machine	87.4% accuracy, 84.0% sensitivity, 88.3% specificity
Pardoel et al. [24]	11 PD patients	machine learning	4 IMUs (L/R shanks, thighs) and plantar pressure sensors	Random Undersampling-boosted decision-tree ensemble model	76.4% sensitivity, 86.2% specificity
Zhang et al. [25]	12 PD patients	machine learning	1 IMU (lower back)	AdaBoost	82.7% accuracy
Demrozi et al. [26]	10 PD patients	machine learning	Daphnet dataset (3 IMUs: ankle, hip, knee)	kNN	94.1% sensitivity, 97.1% specificity
Abbasi et al. [27]	10 PD patients	deep learning	Daphnet dataset (3 IMUs: ankle, hip, knee)	Bidirectional LSTM (with ensemble learning and bottleneck attention module)	99.88% accuracy, 99.99% sensitivity, 98.89% specificity
Wang et al. [28]	10 PD patients	deep learning	Daphnet dataset (3 IMUs: ankle, hip, knee)	multi-channel time-series neural network; intra/inter-channel transformer; attention mechanism	96.21% accuracy, 78.64% precision, 99.13% specificity
Sun et al. [29]	45 PD patients	deep learning	Video recordings (OpenPose annotations)	higher order (3rd-order) polynomial transformer (with self-attention mechanism)	86.1% accuracy, sensitivity: 86.6%, specificity: 84.7%, AUC: 0.92
Yang et al. [30]	12 PD patients	deep learning	5 IMUs (pelvis, L/R tibias, tali)	temporal CNN	0.63 F1-score
Park et al. [31]	12 PD patients	deep learning	Pedar shoe insole system (big toe, forefoot, heel)	temporal CNN	99% accuracy, 68% precision, 88% sensitivity, 90% specificity, 0.76 F1-score
Klaver et al. [32]	70 PD patients	deep learning	combined 4 datasets (7 IMUs: lower back, L/R upper legs, lower legs, feet)	CNN	85% sensitivity, 68% specificity, 0.86 AUC
Borzi et al. [33]	59 PD patients	deep learning	Daphnet dataset (3 IMUs: ankle, hip, knee), ADL dataset (1 IMU: lower back), REMPARK dataset (1 IMU: L waist)	CNN	95.6% sensitivity, 92.9% specificity, AUC: 0.979, 0.621 F1-score
Hu et al. [34]	21 PD patients	deep learning	Footstep pressure sensor gait mat	Adversarial spatial-temporal network	75.7% accuracy, 0.847 AUC
Hou et al. [35]	12 PD patients	deep learning	electroencephelography, 2 IMUs (L/R tibias), and EMG (L/R tibias)	CNN	81% sensitivity, 88% specificity, 0.85 AUC
Borzi et al. [36]	118 PD patients and 21 healthy elderly subjects	deep learning	REMPARK dataset (1 IMU: L waist), 6 MWT (1 IMU: lower back), ADL dataset (1 IMU: lower back),	CNN	0.877 sensitivity, 0.883 specificity, 0.830 F-1 score, 0.946 AUC
Kwon et al. [37]	57 PD patients	deep learning	3D motion capture	attention-based adaptive graphical convolutional network	0.962 F1-score
Shi et al. [38]	63 PD patients	deep learning	2 IMUs (L/R ankles)	CNN	0.915 F1-score, 90.7% Geometric mean score
O’Day et al. [39]	7 PD patients	deep learning	3 IMUs (lower back, L/R ankles)	CNN	0.83 AUC
Filtjens et al. [40]	14 PD patients	deep learning	3D motion capture	CNN (with layer-wise relevance propagation)	86.8% accuracy, 82.1% sensitivity, 88.9% specificity
Shi et al. [41]	67 PD patients	deep learning	3 IMUs (lower back, L/R ankles)	2DCNN	89.2% accuracy, 82.1% sensitivity, 96% specificity, 88.8% Geometric mean score
Pardoel et al. [42]	11 PD patients	machine learning	plantar pressure data	feature selection (Relief-F) followed by ensemble decision tree classifiers	77.3% sensitivity,
					82.9% specificity, 94.2% FOG episodes identified, 0.8 s average prediction time before FOG onset
Bikias et al. [43]	11 PD patients	deep learning	CuPiD dataset (1 IMU: wrist)	CNN	86% sensitivity, 90% specificity
Mostafa et al. [44]	24 PD patients	deep learning	Daphnet dataset (3 IMUs: ankle, hip, knee), A.T. Still dataset (6 IMUs: sternum, lower back, L/R wrists, ankles)	LSTM + CNN	Ankle data: 98.5% ± 0.3% accuracy, 98.5% ± 0.3% precision, 98.5% ± 0.3% sensitivity, 97.9% ± 0.6% accuracy
Shalin et al. [45]	11 PD patients	deep learning	plantar pressure data	LSTM	FOG detection: 82.1% sensitivity, 89.5% specificity; FOG prediction: 72.5% sensitivity, 81.2% specificity
Dvorani et al. [46]	16 PD patients	machine learning	1 IMU (foot)	Support vector machine	81.6 ± 7.7% sensitivity, 70.3 ± 18.4% specificity, 0.828 ± 0.071 AUC
Miao et al. [47]	10 PD patients	deep learning	Daphnet dataset (3 IMUs: ankle, hip, knee)	deep residual network (ResNet)	85.7% sensitivity, 94.0% specificity, 89.7% Geometric mean score
Pardoel et al. [48]	11 PD patients	machine learning	4 IMUs (L/R lower thighs, ankles) and plantar pressure	feature selection (Relief-F) followed by decision tree ensemble	FOG detection: 80.2% sensitivity, 86.3% specificity, 72.6% precision; FOG prediction: 72.2% sensitivity, 83.6% specificity, 60.2% precision
Sigcha et al. [49]	21 PD patients	deep learning	1 IMU (waist)	CNN + LSTM	87.1% sensitivity, 87.1% specificity, 87.1% Geometric mean score, 0.939 AUC
Li et al. [50]	10 PD patients	deep learning	Daphnet dataset (3 IMUs: ankle, hip, knee)	LSTM (with squeeze and excite block and self-attention mechanism)	0.927 sensitivity, 0.952 specificity, 0.976 AUC
Liu et al. [51]	24 PD patients	machine learning	7 IMUs (waist, L/R calves, thighs, feet)	Light GBM	88.62% accuracy, 87.69% sensitivity, 89.29% specificity, 0.8849 AUC
Hu et al. [52]	45 PD patients	deep learning	video recordings	Graph Sequence RNN	82.5% accuracy, 83.8% sensitivity, 82.3% specificity, 0.90 AUC
Borzi et al. [53]	38 PD patients and 21 elderly subjects	machine learning	1 IMU (waist)	Support vector machine	98.3% accuracy, 95.4% sensitivity, 98.8% specificity, 92.8% precision
Reches et al. [54]	57 PD patients	machine learning	3 IMUs (lower back, L/R ankles)	random forest	85.0% ± 10.0% accuracy, 84.1% ± 22.3% sensitivity, 83.4% ± 12.2% specificity, 0.93 AUC
Shalin et al. [55]	11 PD patients	deep learning	plantar pressure data collected from pressure sensor arrays	CNN	94.3% sensitivity, 95.1% specificity
Filtjens et al. [56]	15 PD patients	deep learning	3D motion capture	TCN	Initial contact model: 0.997 F1-score
					End contact model: 0.999 F1-score
Martínez-Villaseñor et al. [57]	10 PD patients	machine learning	Daphnet dataset (3 IMUs: ankle, hip, knee)	Artificial hydrocarbon network	88% accuracy, 88% sensitivity, 88% specificity, 0.88 F1-score
Unni et al. [58]	9 PD patients	machine learning	stepping from force plates	Random Forest	Values not directly reported
Ezhilarasi et al. [59]	12 PD patients	deep learning	2 IMUs (L/R shins)	Faster Mask Region-Based CNN	92% sensitivity, 91% specificity, 0.92 AUC, 95.3% Geometric mean score
Kumar et al. [60]	122 PD patients	deep learning	TDCSFOG dataset (1 IMU: lower back),	1D-CNN with attention mechanism	0.8857 AUC
			DeFOG dataset (1 IMU: lower back)		
Kondo et al. [61]	16 PD patients	deep learning	object tracking and 3D pose estimation	1D CNN + LSTM	93.2% accuracy, 88.8% sensitivity, 97.9% specificity, 97.9% precision, 0.931 F1-score
Pardoel et al. [62]	17 PD patients	machine learning	plantar pressure data	decision tree ensemble with random undersampling boosting (RUSBoosting)	77.42% sensitivity, 80.39% specificity
Yang et al. [63]	18 PD patients	deep learning	5 IMUs (pelvis, L/R tali, tibias)	1D TCN	0.78 F1-score
Banu et al. [64]	10 PD patients	machine learning	Daphnet dataset (3 IMUs: ankle, hip, knee)	CNN	100% accuracy, 100% sensitivity, 1.00 F1-score
Bacanin et al. [65]	93 PD patients	deep learning	plantar pressure data	gated recurrent unit (RNN) with crayfish optimization algorithm	87.0% accuracy, 90.3% precision, 82.3% sensitivity, 86.1% F1 score
Peppes et al. [66]	10 PD patients	deep learning	Daphnet dataset (3 IMUs: ankle, hip, knee)	Generative Adversarial Network	90.66% accuracy
Dimoudis et al. [67]	45 PD patients	deep learning	Daphnet dataset (3 IMUs: ankle, hip, knee), IMU dataset (1 IMU: leg)	inception CNN with squeeze and excite block	98% sensitivity, 99% specificity, 0.972 F1-score, AUC: 0.986
Li et al. [68]	32 PD patients	deep learning	2 IMU accelerometers (L/R ankles) and force-sensitive insoles	LSTM + CNN with squeeze and excite block and attention mechanism	feature-fusion-weighted model: 96.3% accuracy, 92.4% sensitivity, 98.3% specificity, 0.943 F1-score
Önder et al. [69]	10 PD patients	machine learning	Daphnet dataset (3 IMUs: ankle, hip, knee)	Gradient boosted decision trees	92.7% accuracy, 66.7% sensitivity, 96.6% specificity
Koltermann et al. [70]	11 PD patients	deep learning	2 IMU accelerometers (L/R ankles)	CNN	Not directly reported
Sadiq et al. [71]	23 PD patients	deep learning	Daphnet dataset (3 IMUs: ankle, hip, knee), PhysioNet dataset (ground reaction forces)	CNN + LSTM with attention mechanism	98.74% accuracy, 99.25% precision, 99.38% sensitivity, 96.18% specificity, 0.9930 F1-score, 1.00 AUC
Mekruksavanich et al. [72]	10 PD patients	deep learning	Daphnet dataset (3 IMUs: ankle, hip, knee)	CNN with squeeze and excite mechanism	95.66% accuracy, 95.58% precision, 95.66% recall, 95.56% F1-score
Ren et al. [73]	12 PD patients	machine learning	7 IMU accelerometers (waist, L/R thighs, ankles, feet), plantar pressure data	random forest	88.09% accuracy, 78.39% sensitivity, 91.66% specificity, 77.58% precision and 0.7798 F1-score
Filtjens et al. [74]	14 PD patients	deep learning	3D motion analysis	multi-stage spatial-temporal graph convolutional network (MSTGCN)	Not directly reported

**Table 2:** Papers stratified by the location of IMUs used in the training dataset. Note: Some papers utilized multiple sensor locations.

IMU Location	Number of Papers	References
Back	10	[18,25,32,33,36,39,41,44,54,60]
Sternum	1	[44]
Waist	7	[17,33,36,49,51,53,73]
Hip/Pelvis	16	[26,27,28,30,33,44,47,50,57,63,64,66,67,69,71,72]
Thigh/Upper legs	6	[24,32,48,51,67,73]
Knees	14	[26,27,28,33,44,47,50,57,64,66,67,69,71,72]
Tibia/Shin/Lower legs/Shank/Calves	9	[21,23,24,30,32,35,51,59,63]
Ankle/talus	25	[16,26,27,28,30,33,38,39,41,44,47,48,50,54,57,63,64,66,67,68,69,70,71,72,73]
Foot	5	[19,32,46,51,73]
Wrist	3	[21,43,44]

## Data Availability

No new data were created or analyzed in this study.

## Supplementary Materials

The following supporting information can be downloaded at: https://www.mdpi.com/article/10.3390/s26072042/s1.

## Abbreviations

The following abbreviations are used in this manuscript:

PD	Parkinson’s disease
FoG	Freezing of gait
ADL	Activities of daily living
IMU	Inertial measurement unit
CNN	Convolutional neural network
LSTM	Long short-term memory
SMOTE	Synthetic minority oversampling
ML	Machine learning
DL	Deep learning
kNN	K nearest neighbors
SVM	Support vector machine
GenAI	Generative Artificial Intelligence
AI	Artificial intelligence
ECoG	Electrocorticography
DBS	Deep brain stimulation
TUG	Timed Up and Go
RNN	Recurrent neural network
LFP	Local field potential
EMG	Electromyography
EEG	Electroencephalography
STN	Subthalamic nucleus
AUC	Area Under the Curve
